# Supplementary material for: Increased association between Epstein-Barr virus EBNA2 from type 2 strains and the transcriptional repressor BS69 restricts EBNA2 activity
Source: PLoS Pathog. 2019 Jul 8;15(7):e1007458. doi: 10.1371/journal.ppat.1007458 (PMC6638984; doi:10.1371/journal.ppat.1007458)
Supplement: S1 Fig — (A) T2 EBNA2348-422 m3 T1 binding analysed as in Fig 2E and 2F. (B) T2 EBNA2348-422 SD + m3 T1 binding analysed as in Fig 5. (PDF) [file ppat.1007458.s001.pdf]

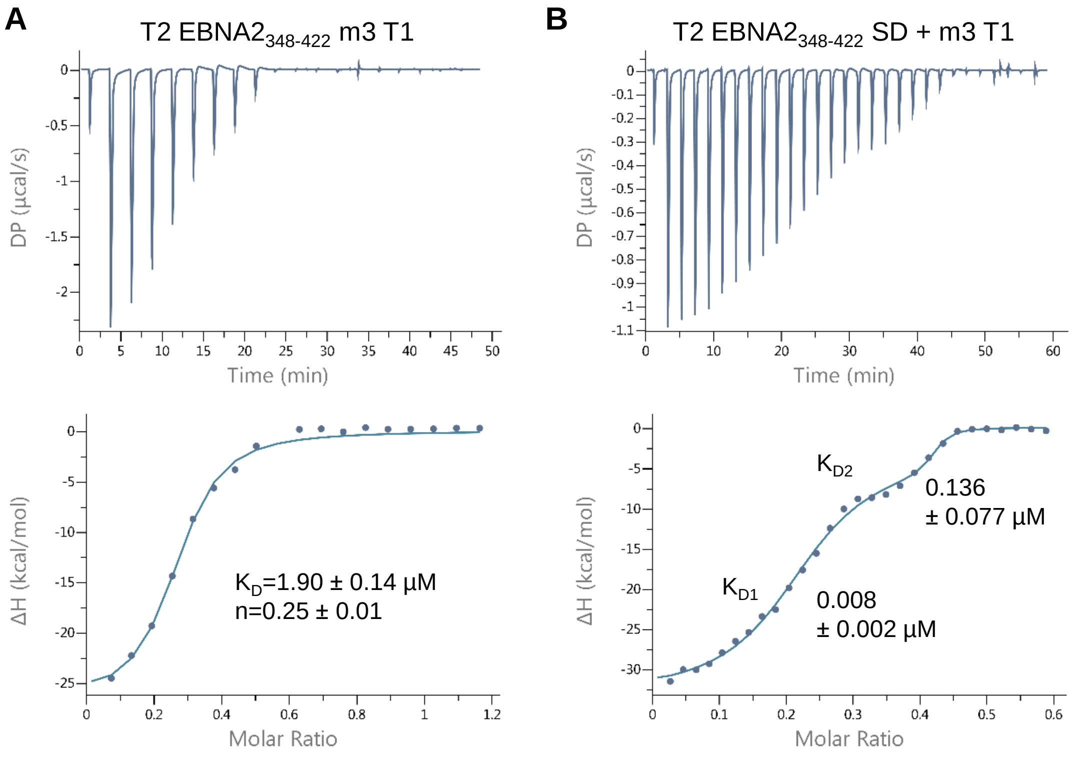

S1 Figure. ITC analysis of the binding of type 2 EBNA2 polypeptides with motif 3 changed from the type 2 sequence (PTLEP) to the type 1 sequence (PSIDP). (A) T2 EBNA2$_{348\text{-}422}$ m3 T1 binding analysed as in Figure 2E-F. (B) T2 EBNA2$_{348\text{-}422}$ SD + m3 T1 binding analysed as in Fig 5.
