# Supplementary material for: Increased association between Epstein-Barr virus EBNA2 from type 2 strains and the transcriptional repressor BS69 restricts EBNA2 activity
Source: PLoS Pathog. 2019 Jul 8;15(7):e1007458. doi: 10.1371/journal.ppat.1007458 (PMC6638984; doi:10.1371/journal.ppat.1007458)
Supplement: S2 Fig — I(q)/I(0)*(q*Rg)2 vs q*Rg. Scattering intensity I (q), scattering vector (q), radius of gyration (Rg). (PDF) [file ppat.1007458.s002.pdf]

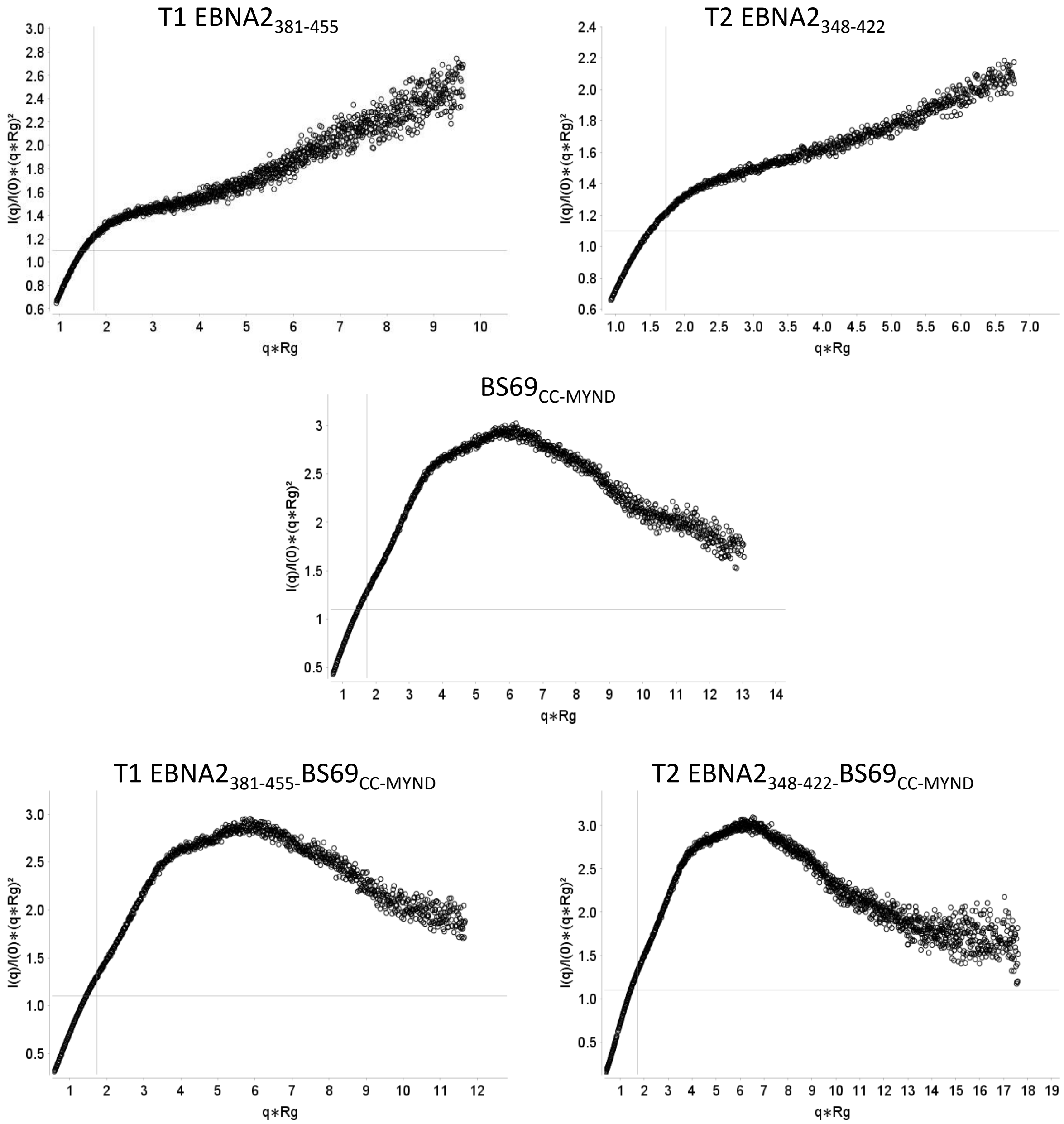

S2 Figure. Normalised (dimensionless) Kratky plots generated using ScÅtter (v3.0 by Robert P. Rambo; Diamond Light Source). $I(q)/I(0)*(q*Rg)^2$ vs q*Rg. Scattering intensity I (q), scattering vector (q), radius of gyration (Rg).
