# Supplementary material for: Increased association between Epstein-Barr virus EBNA2 from type 2 strains and the transcriptional repressor BS69 restricts EBNA2 activity
Source: PLoS Pathog. 2019 Jul 8;15(7):e1007458. doi: 10.1371/journal.ppat.1007458 (PMC6638984; doi:10.1371/journal.ppat.1007458)
Supplement: S3 Fig — (A-C) The SAXS envelopes (grey mesh) were generated by averaging 20 ab-initio models using the DAMMIF programme and further refined with DAMMIN to produce refined dummy atom models (magenta mesh). The maximum dimension (Dmax) and volume were calculated using the ScÅtter programme. In (A) the BS69CC-MYND dimer structure (cyan; PDB ID: 5HDA) was manually docked into the envelope. (D-F) SAXS scattering data (black dots) fitted to the ab initio DAMMIN dummy atom (red line). χ2 values for fitting are shown. (PDF) [file ppat.1007458.s003.pdf]

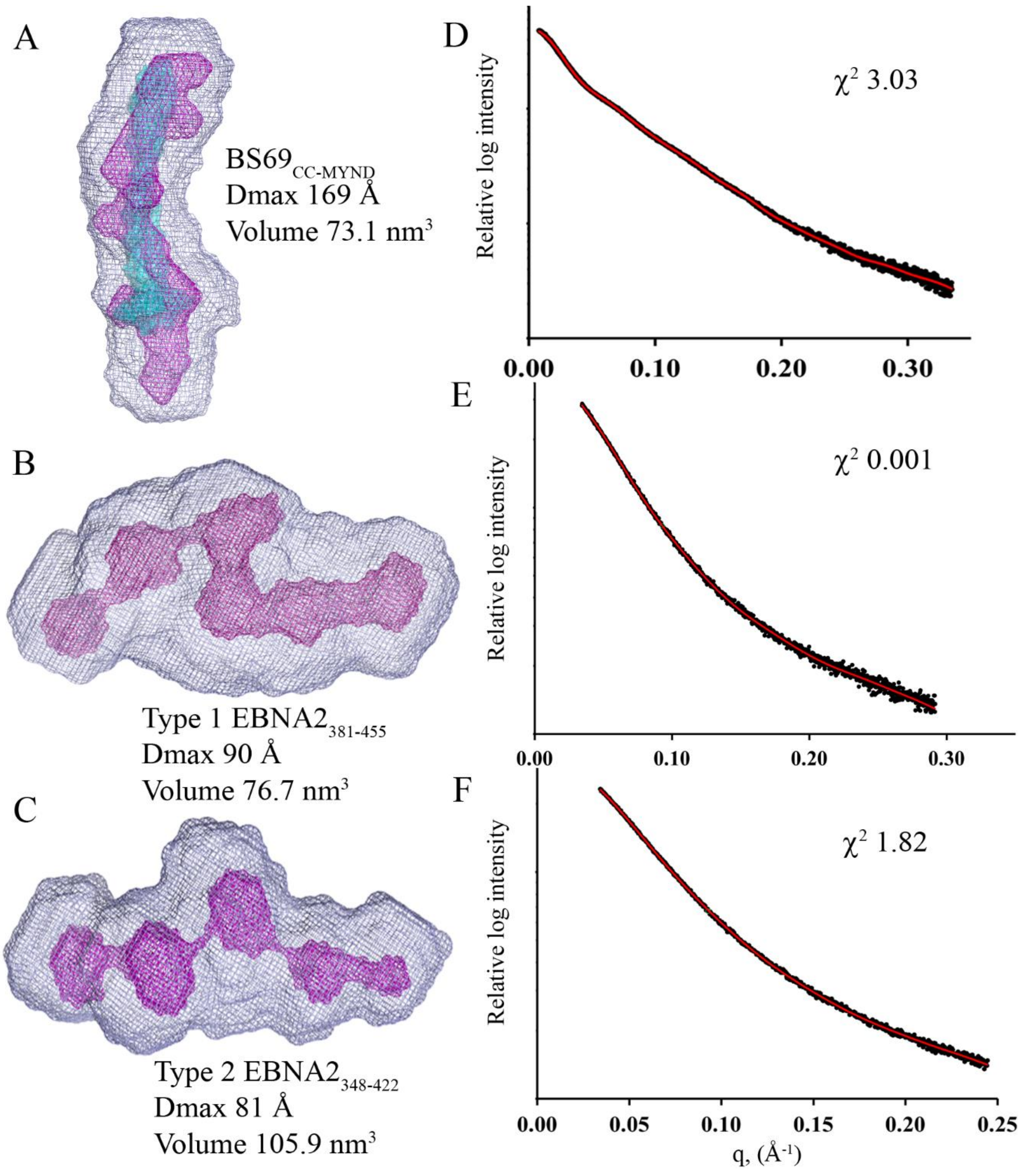

S3 Figure. Solution structures of BS69$_{CC\text{-}MYND}$ and type 1 and type 2 EBNA2 polypeptides determined by SAXS. (A-C) The SAXS envelopes (grey mesh) were generated by averaging 20 *ab-initio* models using the DAMMIF programme and further refined with DAMMIN to produce refined dummy atom models (magenta mesh). The maximum dimension (D_{max}) and volume were calculated using the ScÅtter programme. In (A) the BS69$_{CC\text{-}MYND}$ dimer structure (cyan; PDB ID: 5HDA) was manually docked into the envelope. (D-F) SAXS scattering data (black dots) fitted to the *ab initio* DAMMIN dummy atom (red line). χ^2 values for fitting are shown.
