# Supplementary material for: Increased association between Epstein-Barr virus EBNA2 from type 2 strains and the transcriptional repressor BS69 restricts EBNA2 activity
Source: PLoS Pathog. 2019 Jul 8;15(7):e1007458. doi: 10.1371/journal.ppat.1007458 (PMC6638984; doi:10.1371/journal.ppat.1007458)
Supplement: S4 Fig — (A) One BS69CC-MYND dimer (cyan; PDB ID: 5HDA) and the ab initio model of one type 1 EBNA2381-455 polypeptide (salmon) were manually docked into the ab initio envelope (grey mesh) of the type 1 EBNA2 BS69 complex. (B) SAXS scattering data were fitted to the docked structural complex shown in A and gave a χ2 of 13.33 using FoXS. Graphs show relative log intensity vs scattering vector (q) (upper panel) and the deviation (residual) of the model from the experimental data (lower panel). The hydration parameter (C2) was fixed to 0 to prevent the hydration shell increasing to beyond the maximum limit of 4 to attempt to fit the structure into the envelope. (C) The structural model shown in Fig 4C (two BS69CC-MYND dimers and two type 1 EBNA2381-455 polypeptides) was refitted to the SAXS envelope using FoXS with the C2 value set to 0 for comparison. This gave a similar χ2 (2.59) to that shown in Fig 4A indicating a much better fit to the scattering data. (D) Two BS69CC-MYNDBS69CC-MYND dimers (cyan; PDB ID: 5HDA) and the ab initio model of one type 2 EBNA2348-422 polypeptide (orange) were manually docked into the ab initio envelope (grey mesh). (E) SAXS scattering data were fitted to the docked structural complex shown in D and gave a χ2 of 4.65 using FoXS. Graphs show relative log intensity vs scattering vector (q) (upper panel) and the deviation (residual) of the model from the experimental data (lower panel). The hydration parameter (C2) was fixed to 0 to prevent the hydration shell increasing to high levels to attempt to fit the structure into the envelope. (F) The structural model shown in Fig 4D (three BS69CC-MYND dimers and two type 2 EBNA2348-422 polypeptides) was refitted to the SAXS envelope using FoXS with the C2 value set to 0 for comparison. This gave a similar χ2 (1.47) to that shown in Fig 4B indicating a much better fit to the scattering data. (PDF) [file ppat.1007458.s004.pdf]

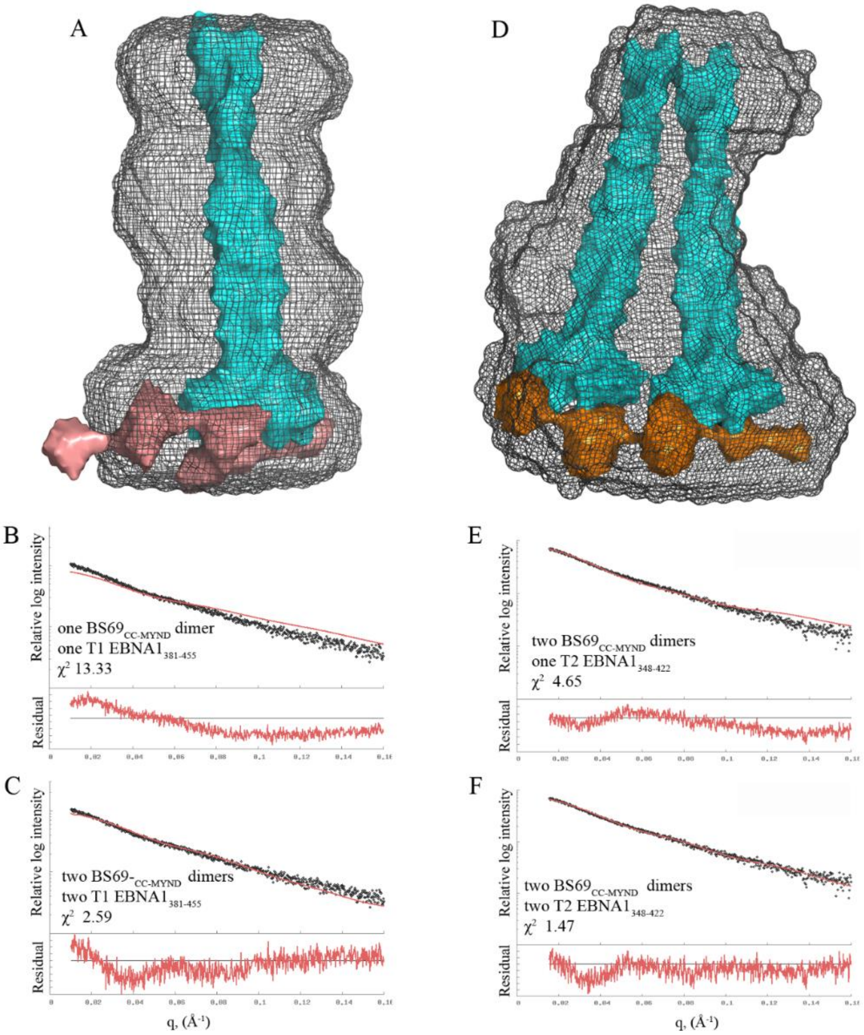

S4 Figure. Alternative models and their respective goodness-of-fit to the experimental SAXS data. (A) One BS69$_{CC\text{-}MYND}$ dimer (cyan; PDB ID: 5HDA) and the *ab initio* model of one type 1 EBNA2$_{381\text{-}455}$ polypeptide (salmon) were manually docked into the *ab initio* envelope (grey mesh) of the type 1 EBNA2 BS69 complex. (B) SAXS scattering data were fitted to the docked structural complex shown in A and gave a χ^2 of 13.33 using FoXS. Graphs show relative log intensity vs scattering vector (q) (upper panel) and the deviation (residual) of the model from the experimental data (lower panel). The hydration parameter (C_2) was fixed to 0 to prevent the hydration shell increasing to beyond the maximum limit of 4 to attempt to fit the structure into the envelope. (C) The structural model shown in Figure 4C (two BS69$_{CC\text{-}MYND}$ dimers and two type 1 EBNA2$_{381\text{-}455}$ polypeptides) was refitted to the SAXS envelope using FoXS with the C_2 value set to 0 for comparison. This gave a similar χ^2 (2.59) to that shown in Figure 4A indicating a much better fit to the scattering data. (D) Two BS69$_{CC\text{-}MYND}$BS69$_{CC\text{-}MYND}$ dimers (cyan; PDB ID: 5HDA) and the *ab initio* model of one type 2 EBNA2$_{348\text{-}422}$ polypeptide (orange) were manually docked into the *ab initio* envelope (grey mesh). (E) SAXS scattering data were fitted to the docked structural complex shown in D and gave a χ^2 of 4.65 using FoXS. Graphs show relative log intensity vs scattering vector (q) (upper panel) and the deviation (residual) of the model from the experimental data (lower panel). The hydration parameter (C_2) was fixed to 0 to prevent the hydration shell increasing to high levels to attempt to fit the structure into the envelope. (F) The structural model shown in Fig 4D (three BS69$_{CC\text{-}MYND}$ dimers and two type 2 EBNA2$_{348\text{-}422}$ polypeptides) was refitted to the SAXS envelope using FoXS with the C_2 value set to 0 for comparison. This gave a similar χ^2 (1.47) to that shown in Fig 4B indicating a much better fit to the scattering data.
