# Supplementary material for: Increased association between Epstein-Barr virus EBNA2 from type 2 strains and the transcriptional repressor BS69 restricts EBNA2 activity
Source: PLoS Pathog. 2019 Jul 8;15(7):e1007458. doi: 10.1371/journal.ppat.1007458 (PMC6638984; doi:10.1371/journal.ppat.1007458)
Supplement: S1 Table — Data show the mean ± standard deviation for three independent experiments (n.d. indicates binding not detected). For peptides n values were fixed to 1. (PDF) [file ppat.1007458.s005.pdf]

	T1 EBNA2 motif 2	T2 EBNA2 motif 2	T1 EBNA2 motif 3	T2 EBNA2 motif 3	T1 EBNA2$_{381-445}$	T2 EBNA2$_{348-412}$	T1 EBNA2$_{381-455}$	T2 EBNA2$_{348-422}$	T2 EBNA2$_{348-422}$ m3 mt	T2 EBNA2$_{348-422}$ m3 T1
**K_D (µM)**	47.7 ± 9.19	176 ± 22.6	n.d.	219 ± 17.7	0.95 ± 0.14	1.21 ± 0.11	0.73 ± 0.07	1.01 ± 0.26	0.59 ± 0.07	1.90 ± 0.14
**n value**	1 (fixed)	1 (fixed)	n.d.	1 (fixed)	0.42 ± 0.02	0.33 ± 0.03	0.38 ± 0.02	0.15 ± 0.01	0.31 ± 0.02	0.25 ± 0.01
**ΔH (kcal/mol)**	-6.80 ± 0.66	-10.8 ± 0.71	n.d.	-19.1 ± 0.78	-26.6 ± 0.53	-22.5 ± 2.48	-23.5 ± 0.46	-53.2 ± 1.68	-25.7 ± 0.89	-27.3 ± 1.23
**-TΔS (kcal/mol)**	0.90 ± 0.54	5.67 ± 0.66	n.d.	14.1 ± 0.78	18.4 ± 0.46	14.4 ± 2.48	15.1 ± 0.42	45.0 ± 1.84	17.2 ± 0.79	19.5 ± 1.27
**ΔG (kcal/mol)**	-5.90 ± 0.11	-5.13 ± 0.07	n.d.	-5.00 ± 0.05	-8.22 ± 0.08	-8.08 ± 0.06	-8.38 ± 0.05	-8.20 ± 0.18	-8.50 ± 0.07	-7.80 ± 0.04
χ^2/degrees of freedom	0.01 ± 0.00	0.01 ± 0.00	n.d.	0.01 ± 0.01	0.04 ± 0.03	0.24 ± 0.06	0.87 ± 0.05	1.55 ± 0.61	0.21 ± 0.06	0.44 ± 0.13

	T1 EBNA2$_{381-455}$ (29 injections)		T2 EBNA2$_{348-422}$ (29 injections)		T2 EBNA2$_{348-422}$ SD (29 injections)		T2 EBNA2$_{348-422}$ SD + m3 mt (29 injections)		T2 EBNA2$_{348-422}$ SD + m3 T1 (29 injections)	
	**Event 1**	**Event 2**	**Event 1**	**Event 2**	**Event 1**	**Event 2**	**Event 1**	**Event 2**	**Event 1**	**Event 2**
**K_D (µM)**	88.5 ± 0.00	3.48 ± 0.00	0.009 ± 0.004	0.091 ± 0.024	0.008 ± 0.003	0.200 ± 0.026	0.005 ± 0.001	0.094 ± 0.028	0.008 ± 0.002	0.136 ± 0.077
**n value**	0.16 ± 0.07	0.30 ± 0.14	0.23 ± 0.01	0.21 ± 0.00	0.22 ± 0.01	0.20 ± 0.00	0.20 ± 0.00	0.19 ± 0.01	0.20 ± 0.01	0.20 ± 0.08
**ΔH (kcal/mol)**	27.8 ± 73.5	-20.3 ± 9.48	-34.1 ± 0.14	-1.59 ± 0.40	-22.7 ± 0.29	-9.62 ± 1.32	-31.7 ± 2.14	-7.65 ± 1.89	-32.1 ± 1.12	-4.82 ± 1.69
**-TΔS (kcal/mol)**	-35.8 ± 63.4	12.0 ± 11.4	23.1 ± 0.14	-8.04 ± 0.25	11.6 ± 0.31	0.47 ± 1.33	20.4 ± 2.24	-1.95 ± 2.10	21.0 ± 0.95	-4.62 ± 2.01
**ΔG (kcal/mol)**	-8.06 ± 4.16	-8.37 ± 1.86	-11.0 ± 0.28	-9.62 ± 0.16	-11.0 ± 0.21	-9.14 ± 0.08	-9.60 ± 0.21	-11.3 ± 0.12	-11.1 ± 0.17	-9.44 ± 0.35
χ^2/degrees of freedom	0.56 ± 0.14		0.23 ± 0.04		0.27 ± 0.13		0.10 ± 0.04		0.39 ± 0.29	

S1 Table. Data obtained from Isothermal calorimetry analysis of EBNA2 peptides and polypeptides binding to BS69$_{CC-MYND}$. Data show the mean ± standard deviation for three independent experiments (n.d. indicates binding not detected). For peptides n values were fixed to 1.
