# Supplementary material for: Increased association between Epstein-Barr virus EBNA2 from type 2 strains and the transcriptional repressor BS69 restricts EBNA2 activity
Source: PLoS Pathog. 2019 Jul 8;15(7):e1007458. doi: 10.1371/journal.ppat.1007458 (PMC6638984; doi:10.1371/journal.ppat.1007458)
Supplement: S3 Table — (PDF) [file ppat.1007458.s007.pdf]

Sample	$BS69_{CC\text{-}MYND}$	T1 $EBNA2_{381\text{-}455}$	T2 $EBNA2_{348\text{-}422}$	$BS69_{CC\text{-}MYND}$ T1 $EBNA2_{381\text{-}455}$	$BS69_{CC\text{-}MYND}$ T2 $EBNA2_{348\text{-}422}$
**Data collection parameters**					
Beam line	B21	B21	B21	B21	B21
Beam geometry (mm^2)	0.25 x 0.25	0.25 x 0.25	0.25 x 0.25	0.25 x 0.25	0.25 x 0.25
Wavelength (nm)	0.09	0.09	0.09	0.09	0.09
s range (nm^{-1})	0.03-0.39	0.03-0.39	0.03-0.39	0.03-0.39	0.03-0.39
Exposure time (s)	3/frame	3/frame	3/frame	3/frame	3/frame
Temperature (K)	293	293	293	293	293
**Structural parameters**					
Rg (nm) [from Guinier]	5.0 ± 0.64	2.7 ± 0.44	2.7 ± 0.79	4.7 ± 0.55	4.7 ± 1.27
Rg (nm) [from P(r)]	5.3 ± 0.88	2.87 ± 0.38	3.0 ± 0.30	4.9 ± 0.83	4.8 ± 2.73
Dmax (nm)	16.9	9.0	8.1	14.9	15.3
Porod volume estimate (nm^3)	73.14	76.67	105.93	165.78	239.07
χ^2 value from DAMMIN	1.33	1.59	1.78	1.40	1.00

S3 Table. SAXS data for BS69 and EBNA2 polypeptides and complexes.
